# Are People Optimistically Biased about the Risk of COVID-19 Infection? Lessons from the First Wave of the Pandemic in Europe

**DOI:** 10.3390/ijerph19010436

**Published:** 2021-12-31

**Authors:** Kathleen McColl, Marion Debin, Cecile Souty, Caroline Guerrisi, Clement Turbelin, Alessandra Falchi, Isabelle Bonmarin, Daniela Paolotti, Chinelo Obi, Jim Duggan, Yamir Moreno, Ania Wisniak, Antoine Flahault, Thierry Blanchon, Vittoria Colizza, Jocelyn Raude

**Affiliations:** 1 Unite des Virus Emergents, Institut de Recherche pour le Développement 190, Institut National de la Santé Et de la Recherche Médicale (INSERM) 1207, Health, Aix-Marseille University, 13009 Marseille, France; Jocelyn.Raude@ehesp.fr; 2 École des Hautes Études en Santé Publique (EHESP) French School of Public Health, 35043 Rennes, France; 3 Institut National de la Santé Et de la Recherche Médicale (INSERM), Institut Pierre Louis d’Épidémiologie et de Santé Publique (IPLESP), Sorbonne Université, F-75012 Paris, France; marion.debin@iplesp.upmc.fr (M.D.); cecile.souty@iplesp.upmc.fr (C.S.); caroline.guerrisi@iplesp.upmc.fr (C.G.); clement.turbelin@iplesp.upmc.fr (C.T.); thierry.blanchon@iplesp.upmc.fr (T.B.); vittoria.colizza@inserm.fr (V.C.); 4 Laboratoire de Virologie, Unité de Recherche 7310, Université de Corse, 20250 Corte, France; falchi_a@univ-corse.fr; 5 Santé Publique France, 94410 Saint Maurice, France; Isabelle.BONMARIN@santepubliquefrance.fr; 6 Istituto per l’Interscambio Scientifico, ISI Foundation, 10126 Turin, Italy; daniela.paolotti@isi.it; 7 Public Health England, London SE1 8UG, UK; Chinelo.Obi@phe.gov.uk; 8 School of Computer Science, National University of Ireland, H91 TK33 Galway, Ireland; james.duggan@nuigalway.ie; 9 Institute for Biocomputation and Physics and Complex Systems, University of Zaragoza, 50001 Zaragoza, Spain; yamir.moreno@gmail.com; 10 Faculty of Medicine, Institute of Global Health, University of Geneva, 1202 Geneva, Switzerland; Ania.Wisniak@unige.ch (A.W.); Antoine.Flahault@unige.ch (A.F.)

**Keywords:** risk perception, optimism bias, unrealistic optimism, COVID-19, pandemic, Europe

## Abstract

Unrealistic optimism, the underestimation of one’s risk of experiencing harm, has been investigated extensively to understand better and predict behavioural responses to health threats. Prior to the COVID-19 pandemic, a relative dearth of research existed in this domain regarding epidemics, which is surprising considering that this optimistic bias has been associated with a lack of engagement in protective behaviours critical in fighting twenty-first-century, emergent, infectious diseases. The current study addresses this gap in the literature by investigating whether people demonstrated optimism bias during the first wave of the COVID-19 pandemic in Europe, how this changed over time, and whether unrealistic optimism was negatively associated with protective measures. Taking advantage of a pre-existing international participative influenza surveillance network (*n* = 12,378), absolute and comparative unrealistic optimism were measured at three epidemic stages (pre-, early, peak), and across four countries—France, Italy, Switzerland and the United Kingdom. Despite differences in culture and health response, similar patterns were observed across all four countries. The prevalence of unrealistic optimism appears to be influenced by the particular epidemic context. Paradoxically, whereas absolute unrealistic optimism decreased over time, comparative unrealistic optimism increased, suggesting that whilst people became increasingly accurate in assessing their personal risk, they nonetheless overestimated that for others. Comparative unrealistic optimism was negatively associated with the adoption of protective behaviours, which is worrying, given that these preventive measures are critical in tackling the spread and health burden of COVID-19. It is hoped these findings will inspire further research into sociocognitive mechanisms involved in risk appraisal.

## 1. Introduction

The current coronavirus (SARS-CoV-2) pandemic has highlighted the importance of personal protective behaviour in limiting the spread of disease [1,2,3], as well as the reluctance of some individuals and groups to comply with public health recommendations, such as social distancing [3,4,5,6]. Fundamental to the adoption of and adherence to such behaviours are a range of psychological and social factors, in particular a person’s perception of risk [7,8,9,10], which is thought to play a key role in combating twenty-first-century global health threats such as the COVID-19 pandemic [11,12,13,14,15]. Research into behavioural responses to health threats suggests that risk perception is a major determinant in adopting measures to prevent disease [9,16,17,18,19,20]. Failure to appraise the risk of a particular disease in a realistic way not only increases the likelihood of experiencing ill health, it may also lead to disease proliferation, overloaded public health facilities, serious illness and death [4,5,21]. As perceived risk increases, so too does the motivation to adopt precautionary measures [22,23,24,25,26,27,28,29,30,31,32,33]. However, studies of chronic health threats repeatedly reveal that people are likely to demonstrate optimism bias, also called unrealistic optimism, when faced with a health threat, i.e., people tend to underestimate their likelihood of experiencing a negative event, notably when compared with someone else [10,22,34,35]. For instance, Weinstein [23] discovered that when college students were asked to estimate their likelihood of suffering from 45 different health problems of varying severity compared with that of their fellow students, they significantly rated themselves as less likely than their counterparts to suffer from 34 of these health issues. Furthering this research, Weinstein, Klotz and Sandman [36] found that following reports of hazardous levels of radon in residential areas of the US, residents of the areas concerned believed that they were less at risk of radon contamination than their neighbours. Turning his attention to smoking, Weinstein [24] observed that smokers believed themselves to be less at risk than other smokers of encountering related negative health outcomes. Underestimation of one’s personal risk, as compared with that of others has also been observed for a variety of chronic health issues (e.g., [10,22,23,24,37,38]), ranging from mild to severe health threats [20,39,40]. This bias in risk judgement is believed to inhibit engagement in health-protective behaviours [41,42,43]. As the world faces risk, tragedy and economic hardship associated with the current COVID-19 pandemic, understanding how the spread of this disease affects the perceived risk of personal infection and vice versa, as well as its impact on subsequent behaviour, may provide valuable insight into not only the current pandemic, and subsequent waves thereof, but also future epidemics. 

Prior research into optimism bias has generally focused on either absolute or comparative unrealistic optimism. Absolute optimism bias, a person’s belief that s/he will have a more favourable outcome than is statistically likely as measured objectively using epidemiological data, has been observed in people’s underestimation of their chances of a negative health outcome [30,40,44]. 

Comparative unrealistic optimism, an individual’s underestimation of his or her likelihood of contracting a particular disease as compared with someone else, has been measured using either a direct or indirect approach. The former involves a single-scale participant rating as being more, as, or less likely than another person to contract a particular disease [10,23,45,46]. Critics of the direct method propose that the use of a single scale leads participants to attend more to their personal risk, rather than making a true comparison. Using an indirect approach, involving two distinct scales, one measuring participants’ estimations of their likelihood of encountering a particular health problem, and a second scale rating the likelihood that someone else might encounter that same health problem, is preferred as it focuses participant attention independently on either their personal risk or on that of others [22,26,30,32,47,48]. This indirect approach is therefore adopted in the current study. The terms “optimism bias” and “unrealistic optimism” are used interchangeably. The current investigation explores whether and how levels of absolute and comparative unrealistic optimism vary over three times and across four countries during the first wave of the COVID-19 pandemic.

As investigations into the influence of optimism bias on behaviour have been largely cross-sectional and retrospective [47,49], it is impossible to know at what stage people are in their risk appraisal or unrealistic optimism. An individual may initially perceive risk to be high, engage in protective behaviours, and once these measures have been taken, believe that risk is reduced, therefore demonstrating a somewhat justifiable, not-so-unrealistic optimistic bias [32,50]. One might even question whether such optimism is indeed unrealistic. Moreover, individuals who appraise their risk as high are more likely to engage in protective behaviour, thereby reducing their risk and increasing optimism [33]. It is therefore impossible to tell whether unrealistic optimism is the cause or consequence of protective behaviour [32,43,51]. Investigating changes in unrealistic optimism throughout the first wave of the pandemic may reveal interesting trends in risk perception and behaviour across the four countries, as they reacted to different epidemic, societal and government responses, and may ultimately contribute to informing targeted public health messages.

It should be noted that misgivings regarding optimism bias research have been directed towards inappropriate design and wording of questionnaires which could unintentionally influence participant responses [26,52]. Numeracy difficulties involving risk estimates reliant upon participants’ grasp of probability, particularly small probabilities, odds proportions and ratios, may not always be accurate [21,53,54,55], thereby contributing to biases in appraising risk and therefore unrealistic optimism [56]. To overcome these measurement problems, the current study avoids participant estimations reliant upon probabilities and ratios.

Whilst associations have been found between optimism bias and protective behaviour regarding chronic disease or health issues (e.g., [23,24,25,45,50,57,58,59]), few empirical studies to date have focused on these interrelated, dynamic factors during an evolving epidemic setting, and even fewer during a global pandemic of an emerging, highly infectious disease. Little is therefore known about how people adjust and update their risk-related beliefs in a rapidly changing epidemiological environment [60]. The current study seeks to address this gap in the literature by exploring variations in the impact of optimism bias on risk appraisal and behavioural response at three stages during the first wave of the 2020 COVID-19 pandemic. Building upon research suggesting that levels of optimism bias may vary across countries and epidemiological settings [21,61,62], this study spans four countries—France, Italy, Switzerland and the United Kingdom (UK), as Europe was at the pandemic epicentre—to investigate the following questions at three stages during the first wave of the pandemic:

1. Is people’s perceived risk of infection absolutely or comparatively skewed? If so, do levels of optimism vary at each of the three (pre-, early and peak) pandemic stages within and across countries?
2. Are there differences in comparative optimism among subpopulations?
3. Is comparative optimism negatively associated with protective behaviours?

Through this research, we wish to gain a better understanding of how an evolving epidemiological context influences optimism bias in risk judgments and subsequent behavioural responses, and hope this will inform future campaigns reliant upon and targeting behaviour change during the course of a pandemic to reduce the spread and exponential death toll from emerging infectious diseases, such as that of COVID-19.

### 1.1. Optimism Bias in an Epidemic Context

Focusing predominantly on chronic diseases, research into optimism bias to date has largely neglected contextual factors, concentrating instead upon individual factors that contribute to risk appraisal and optimism bias [43,63]. The novel nature of the current, constantly evolving COVID-19 pandemic constitutes a dynamic contextual factor that may well impact upon biases, optimistic or otherwise, in risk judgements and resultant engagement in protection [3,12,21]. Understanding such biases is critical in combatting this disease and the associated health, economic and psychological burden. 

Of the few investigations of optimism bias in an epidemic setting, two found similar patterns of unrealistic optimism as those for chronic health threats concerning vaccination intention among US college students during the 2009–2010 H1N1 pandemic [64,65]. In a further H1N1 study, optimism bias was found to be pervasive but had no significant effect on behaviour [66]. However, contrasting patterns of optimism bias and behaviour were found in Rudisill’s [67] research during the 2009 H1N1 pandemic in the UK, in which the majority of participants (58%) did not show comparative unrealistic optimism, and of those 42% who did, unrealistic optimism was not associated with precautionary behaviour. Overall, greater perceived personal risk and second-hand experience, rather than comparative unrealistic optimism, were associated with protective measures, leading the author to propose that uncertainty, inherent in an epidemic, influences risk perception and subsequent behaviour.

In their cross-sectional study exploring the dynamic nature of risk perception and behaviour towards the unfolding 2009 H1N1 pandemic in Beijing, Xu and Peng [68] found that while participants demonstrated optimism bias, associations between risk appraisal and preventive measures were unstable over the course of the epidemic across three intervals. Although behaviours requiring little effort were generally maintained, there was a negative association between risk judgement and behaviour a year prior to the pandemic (T1). This association changed, becoming positive at the beginning of the outbreak (T2). However, at the height of the pandemic (T3), and despite rapidly increasing new case numbers, no association between risk perception and behaviour was found, perhaps due to the mild nature of H1N1 symptoms and the commencement of community vaccination campaigns. 

A related investigation targeting the dynamic nature of influenza vaccine uptake among Italian health care workers over three consecutive seasons also suggested that altered risk perceptions, that serve to diminish risk, play a key role in vaccination intention, or rather lack thereof [69]. The authors highlight the urgent need for further research in this field. Building and expanding upon this research, the current study, also conducted in an evolving epidemic setting, seeks to examine changes in risk perception among members of the general public during the course of the COVID-19 first wave. 

Previous cross-cultural research into optimism bias and protective behaviour during an epidemic outbreak of a genetically similar coronavirus, severe acute respiratory syndrome (SARS) [70], found that whereas both Canadian and Chinese participants displayed comparative optimism, the latter displayed greater levels of thereof, as well as increased engagement in protective behaviours, than did their Canadian counterparts. Moreover, both populations overestimated the absolute risk of becoming infected, as compared with actual prevalence [21]. Research at two different stages of the 2003 Hong Kong SARS outbreak found that despite people’s judging the risk of infection to have decreased by the second epidemic phase, they nonetheless maintained their protective behaviours [71]. Although not specifically investigating optimism bias, risk perceptions nonetheless changed rapidly during the epidemic, emphasising the pivotal role played by the epidemic context. In a further investigation of a hypothetical future SARS epidemic, De Zwart et al. [61] found people demonstrated a pessimistic bias towards a new risk they saw as uncontrollable; however, these data were gathered two years after the SARS outbreak. Each of these authors urged future researchers to investigate biases in risk judgement as an epidemic or pandemic evolves, in order to provide findings of greater contextual relevance.

Early research into the current COVID-19 pandemic suggests that people were comparatively optimistically biased regarding their likelihood of becoming infected during the initial stages of the disease outbreak in the UK, the US, Germany, France, Italy and Switzerland [2,61]. A similar trend was also observed in student populations in Iran, Kazakhstan and Poland [72,73]; however, these latter findings must be interpreted with caution as undergraduate students are by no means representative of the general population, and particularly as far as COVID-19 is concerned, are far less at risk of developing severe or life-threatening forms than the rest of the adult population [14]. Dolinksi et al. [73] nonetheless reinforce the urgent need for research into optimism bias and protective behaviour in light of the COVID-19 pandemic. Our study endeavours to address this issue, as well as extending the initial research to the general population and expanding it across four countries. 

Investigating changes in risk perception and behaviour during the first five days of the COVID-19 outbreak in the US, Wise et al. [3] found that whereas most participants’ awareness and adoption of protective measures increased, they nonetheless remained comparatively optimistic. Alarmingly, an extremely comparatively optimistic subgroup of participants, demonstrating low engagement in protective measures, was identified. Such a subgroup, if representative of the actual population, could lead to the rapid propagation of this highly infectious disease, reinforcing the need for further research to be conducted in this area.

Past investigations of the evolutive nature of risk perception and protective behaviour, predominantly through mathematical modelling, have highlighted the importance of and inherent difficulty in measuring and analysing shifts that occur, both cognitive and behavioural, in response to a constantly evolving epidemiological setting, as well as the need to expand upon research in this field [12,74,75,76,77,78]. The strength of the current research lies in its assessment of dynamic cognitive and behavioural variations at three intervals during the rapidly evolving first wave of the global COVID-19 pandemic.

### 1.2. Current Epidemiological Context

Belonging to the family of coronaviruses, including SARS, Middle Eastern Respiratory Syndrome (MERS) and the common cold, severe acute respiratory syndrome coronavirus (SARS-CoV-2), results in the highly contagious disease, COVID-19 [14,79,80]. First reported in China in December 2019 [81], it spread rapidly throughout the world and by March 11 2020, was declared a global pandemic by the World Health Organisation [14]. 

Whilst often asymptomatic or resulting in relatively mild symptoms (90% of cases), more severe forms (5–10% of cases) lead to pneumonia and life-threatening disease requiring hospitalisation, and in some cases, death. People over 70 and those with a pre-existing comorbidity are particularly at risk [82,83,84,85]. Transmission is believed to occur via airborne droplets from an infected person during normal social interactions and via contaminated surfaces. The highly contagious nature of COVID-19, combined with new variants and severe cases requiring hospitalisation, rapidly place a huge strain upon health care systems [14,86]. 

These elements illustrate the need for individual adoption of health-protective actions such as barrier measures and physical distancing in order to limit disease spread [86,87]. With no readily available cure for COVID-19, a lengthy vaccine roll-out in Europe [88] and the arrival of new virus variants, the crucial role of individual protective measures is reinforced [2,3,14,80,86,89,90]. Understanding cognitive factors contributing to the engagement in such protective measures is therefore paramount and underpins the current research.

Given that contextual factors may influence biases in risk judgement and protective behaviour adoption [21,91], it is necessary to consider the particular epidemiological setting and government-imposed measures to limit COVID-19 spread in each country. At the time of the first survey (12–21 February 2020), no lockdown measures were in place. The second survey took place as physical distancing and barrier actions were recommended in France, Switzerland and the UK, and Italy’s lockdown had been tightened. The final survey was conducted as Italy, France and the UK were in lockdown and government restrictions had been imposed in Switzerland [92,93,94,95,96]. Throughout this period across Europe, the importance of protective measures in reducing COVID-19 spread and its subsequent health toll was emphasised [14,80,86]. These elements set the research scene for each phase of the current study.

Optimism bias towards chronic illnesses has been frequently associated with a reduction in protective behaviours [21,22,97,98]; however, whether this occurs in an evolving pandemic setting remains unknown. Interrelated contextual factors, such as the novel nature of COVID-19 or its rapid proliferation, may reduce optimism bias [2,3,21,43,71]. The current research seeks to provide valuable insight into variations in biases, optimistic or otherwise, and engagement in protective measures as the epidemic unfolds, in order to foster reflection upon ways in which public health campaigns might best target and motivate people to protect themselves and others in the face of a highly contagious, rapidly spreading emerging infectious disease. 

## 2. Methods

### 2.1. Participants and Procedures

We took advantage of an existing participatory monitoring system, “Influenzanet”, for influenza-like illnesses amongst the general population in Europe [99]. Four countries at the epicentre of the first wave of the pandemic in Europe agreed to conduct a survey investigating risk perception and protective behaviours related to COVID-19. Each country ran its own website, on which people registered and participated voluntarily. Recruitment took place via communications by the media, universities and supporting institutions, social media, promotional events and word of mouth. Participants completed an intake survey covering demographic, geographical, socioeconomic and health-related factors, followed by regular weekly surveys, in which they declared and described the presence or absence of symptoms since their last online report [100,101]. A personal link to a COVID-19 perception questionnaire was sent to “Influenzanet” participants in France, Italy, Switzerland and the UK at three stages (T1 = 12–21 February 2020, T2 = 11–12 March 2020, T3 = 31 March–5 April 2020) during the first wave of the COVID-19 pandemic. There was no time limit on the online survey. Participation rates (available for France only) were 49% for the first survey and 50% for both the second and third surveys. Overall, 12,378 participants fully completed the online questionnaires (9783 French, 913 Italian, 1029 Swiss and 653 British participants). 

Ethical approval by Ethical Review Boards or Committees was sought, in accordance with country-specific regulations and informed consent was obtained from all participants. 

### 2.2. Measures

#### 2.2.1. Risk Perception

Perceived risk of SARS-CoV2 infection was assessed during the course of the epidemic using two validated items adapted from the literature on comparative risk judgment [20]: (1) “What is the risk for yourself of catching this coronavirus in the coming weeks?”, and (2) “In your opinion, out of 100 [name of country] people, how many of them are at risk of catching this coronavirus in the coming weeks?” Participants were asked to rate each item on a discrete percentage scale ranging from 0% to greater than 50%. In the third survey, participants were given the option of answering: “I think I already had this disease” when asked to assess their personal risk of contracting the disease (See Table 1).

#### 2.2.2. Unrealistic Optimism

To determine whether perceived risk of infection was optimistically biased, an indirect measurement method was used, which consists of comparing the infection risk participants estimated for themselves and that for others, measured on two separate scales [26,51]. In our surveys, unrealistic optimism was observed when a person’s own risk of infection was judged as being lower than that of others, whereas pessimism was observed when it was estimated as being higher than that of others [102]. Conversely, participants were realistic when they estimated their personal risk to be no different from that of others. To facilitate the subsequent treatment of the comparative risk judgements, data obtained by comparing these two items were dichotomised with the “unrealistic optimism” category being coded as “1”, and the other options (“pessimism” and “realism”) combined into a “non-unrealistic optimism” category, coded as “0”. In the third survey, people who reported having had the disease were excluded. 

#### 2.2.3. Health-Protective Behaviours

Participants were asked whether they had adopted a variety of behaviours to reduce the risk of COVID-19 infection. At the early stage of the pandemic, only a small number of preventive measures was specifically recommended by the public health authorities, such as the WHO, to prevent infection [14]. Our surveys investigated a larger range of health-protective behaviours than those recommended in international guidelines, and included “wash hands often”, ”wear a face mask”, “avoid touching one’s mouth and nose”, “use a tissue only once when coughing or sneezing”, “avoid public transportation”, “use sanitising hand gel”, “avoid contact with people who look sick”, and “avoid social events”. The possible response to each of these items was “Yes” or “No” (See Table 1).

#### 2.2.4. Sociodemographic and Illness-Related Variables

Participants’ sociodemographic and health-related information, including country, sex, age, education, occupation, household composition, place of residence, personal history of COVID-like symptoms (dates, type of symptoms), and details about diagnosis and treatment, were collected. Participants were also asked whether they were taking medication for at least one of the following conditions: asthma, diabetes, chronic lung disorder besides asthma, heart disorder, kidney disorder or an immunocompromising condition.

### 2.3. Data Analyses

Percentages of unrealistic optimism were calculated for all subgroups of the population and epidemic stages. However, to facilitate data analysis, as well as the presentation of the results, several sociodemographic and health-related variables were re-coded from the original database: *participant age* into three categories (18–34, 35–64, ≥65), “education” into two categories (≤A-level, >A-level), and household characteristics into three categories: “living alone”, “living with one or several children”, “living with adult(s) and without a child”. The “chronic condition” variable included people taking medication for at least one of the following: asthma, diabetes, chronic lung disorder other than asthma, heart disorder, kidney disorder, or an immunocompromising condition.

As the gender-age structure and education distribution of the “Influenzanet” sample differed from those for the general population [103], analyses were reweighted on age group, gender and education. Sociodemographic and education data used for adjustment were obtained from the European Commission [104], the United Nations Statistics Division [105], the French National Institute of Statistics and Economic Studies (Institut National de la Statistique et des Études Économiques, INSEE) [106], the Federal Statistical Office [107], and the Italian National Institute of Statistics (Instituto Nazionale di Statistica) [108]. To compare differences in weighted proportions of unrealistic optimism between participants according to country and stage of the epidemic, chi-square and *p*-values were calculated.

Associations between sociodemographic, health determinants and unrealistic optimism were estimated using logistic regression models. Explanatory variables were first tested in a series of univariate analyses. All covariates were then tested in multivariate analyses, through a random effects mixed model, taking into account the non-independent nature of answers given by the same participant in successive questionnaires. Covariates were selected through a backward step-wise selection. The final model included all covariates associated with a *p*-value below or equal to 0.05.

The effects of unrealistic optimism on protective behaviours were assessed using a series of random effects mixed models adjusted on the participant’s age, sex, level of education, and country. 

Statistical analyses were performed using R software, version 3.4 [109].

## 3. Results

### 3.1. Are Perceived Risks of SARS-CoV-2 Infection Optimistically Biased?

**Figure 1:**
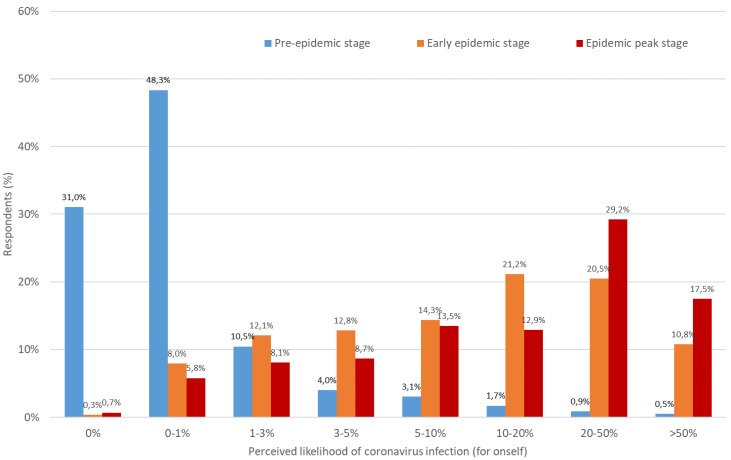
Personal perceived likelihood of becoming infected with COVID-19 at the pre-, early and peak epidemic stages.

**Figure 2:**
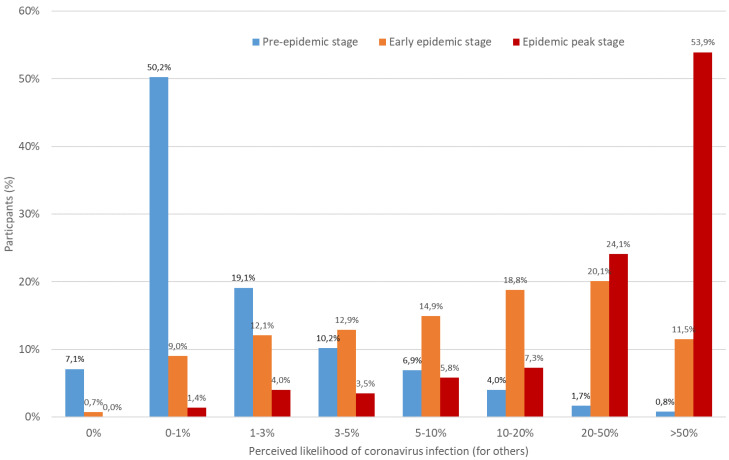
Perceived likelihood of COVID-19 infection for others at the pre, early and peak epidemic stages.

As shown in Figure 1 and Figure 2, perceived risk of SARS-CoV-2 infection for both oneself and for others increased substantially over the course of the epidemic in Europe. Notably, the median value of perceived risk for oneself increased from a range of 0–1% in the pre-epidemic stage to 10–20% in the subsequent stages of the epidemic, while that for others increased from 0% to 1% to greater than 50% during the peak of the epidemic. Retrospectively, it can be established that there was no optimism bias from an absolute perspective in personal risk judgements made by participants, with the exception of the pre-pandemic stage during which a large majority of participants across countries underestimated their personal risk of catching COVID-19. Indeed, at the time of the third survey, according to government statistics, an estimated 5% of people in France [110], Italy [111] and Switzerland [112], and 15% of people in the UK [113] had been infected with COVID-19. Overall, absolute unrealistic optimism tended to disappear among participants as the disease spread in Europe.

There were nonetheless significant differences in ratings of self and other risk perception items over time (Wilcoxon signed rank test value with continuity correction = 274,739, 540,996, and 2,467,056 at T1, T2, and T3, respectively; *p* < 0.001). The results of the comparison between the two items, which allows measurement of unrealistic optimism according to the indirect method, are presented in Table 2. Unexpectedly, it was found that the rate of comparative optimism among participants significantly increased with the proportion of infected individuals over the course of the pandemic. Only French participants’ risk perceptions were characterised by a non-linear relationship between comparative optimism and disease burden (a U-shaped curve), as the rate of optimism decreased between T1 and T2 (-7 percentage points), then substantially increased between T2 and T3 (+18 percentage points). Nevertheless, the rate of unrealistic optimism was at its highest across all four countries as the epidemic reached its first-wave peak in Europe. 

### 3.2. Are There Differences in Comparative Optimism among Subpopulations?

The distribution of unrealistic optimism in comparative risk judgments within a variety of groups and settings is presented in Table 3. Apart from the initial epidemic stage in Europe, optimists were in the majority in all subpopulations and epidemiological contexts (ranging from 49% in Switzerland to 63% in Italy). However, there were considerable differences in the level of unrealistic optimism, depending on participant sociocultural, demographic and health characteristics. As shown in Table 4, a series of regression analyses were performed using binary logistic models, in which unrealistic optimism was treated as the dependent variable and regressed on the various characteristics of participants. Unadjusted odds ratios were the highest for the early epidemic stage (UOR = 2.38, 95% CI = 2.17–2.61, *p* < 0.001) and for the Italian participants (UOR = 1.82, 95% CI = 1.47–2.22, *p* < 0.001). Surprisingly, for the “chronic health condition”, the unadjusted odds ratio was non-significant (UOR = 0.92, 95% CI = 0.84–1.01, *p* = 0.097). As some of these independent variables may be significantly inter-correlated, a multiple regression analysis was performed to assess the robustness of the influence of each of these personal and contextual factors on unrealistic optimism, independent of other predictors. Overall, the results of the multivariate analyses were strongly consistent with those from earlier univariate analyses. Only *household composition* was no longer significant as a predictor of unrealistic optimism.

### 3.3. Is Unrealistic Optimism Associated with the Adoption of Protective Behaviours?

To determine whether the adoption of protective behaviours was significantly associated with comparative unrealistic optimism, a series of logistic regression models were conducted with a variety of protective measures commonly adopted during the pandemic to prevent SARS-CoV-2 infection across countries as a binary variable, serving as the dependent variable. The odds ratios and their 95% confidence intervals after adjustment for participant age, sex, level of education, and country for each preventive measure are shown in Table 5. Unrealistic optimism was significantly and negatively associated with the majority of the health-protective behaviours measured (“wear a face mask”, “avoid touching one’s mouth and nose”, “use sanitising hand gel” and “avoid contact with people who look sick”). Only the recommendations to “wash hands often”, “avoid public transportation” and “use a tissue only once” were followed to a similar extent by all participants (*p* > 0.05).

## 4. Discussion

According to the psychological and behavioural literature, the tendency to underestimate or deny the risk of experiencing harm observed in unrealistic optimists may inhibit the very behaviour change necessary to control or prevent health threats (e.g., [10]). In the context of the COVID-19 epidemic, this concept has provided media observers and scientific experts with an explanation for the delayed and/or inadequate response to the coronavirus threat [114]. Nonetheless, the question remains as to whether the concept of optimism bias accurately captures what was happening in the various countries, as this could affect policies and programs targeting behavioural change. This study takes advantage of a pre-existing international participative network of influenza surveillance to address this question by providing insight into people’s absolute and comparative unrealistic optimism at three stages in an evolving social and epidemiological context. Data from our surveys show that whether people are unrealistically optimistic about the risk of infection from COVID-19 depends to a large extent on when they are asked.

### 4.1. A Paradoxical Trend in Optimism

We found that absolute unrealistic optimism decreased over time, so by the time the first wave of the epidemic reached its peak in Europe, participants were increasingly accurate in estimating their personal risk of catching COVID-19, as compared with epidemiological data. This trend is compatible with a Bayesian approach of risk perception, whereby people update their beliefs in response to new information about the evolving COVID-19 epidemic. However, these same participants became increasingly comparatively optimistically biased, overestimating other people’s likelihood of becoming infected. Thus, although becoming more accurate over time in estimating personal risk, participants nonetheless believed others to be increasingly more at risk than themselves. These findings differ from those of Ji et al. [21], who found that participants demonstrated increasing levels of both absolute and comparative unrealistic optimism bias as the SARS epidemic progressed. Our findings also contrast those of Wise et al. [3], who observed a decrease in comparative unrealistic optimism over the first five days of the US COVID-19 outbreak. Such differences may be attributable to interview timing, as well as the nature of the epidemic or pandemic. Ji et al. [21] conducted interviews one year prior to the SARS outbreak and again at its peak, whereas Wise et al.’s [3] interviews only covered the initial days of the US COVID-19 outbreak. Our findings, on the other hand, spanned the first two months of the coronavirus pandemic in Europe and suggest that the epidemiological stage and context, as well as the very nature of the virus, may play a crucial role in the magnitude of bias in risk perception. 

The generalised inverse pattern over time of decreasing levels of absolute unrealistic optimism, and increasing comparative unrealistic optimism, was observed in each of the four European countries, which is somewhat surprising considering the national variations in health response and culture, suggesting perhaps a more homogeneous trend in risk perception regarding COVID-19 than previously observed for other epidemics. Similar trends in unrealistic optimism towards COVID-19 were observed in Germany, the USA and the UK by Kuper-Smith et al. [62]. In contrast, Chinese participants in Ji et al.’s [21] SARS study were significantly more comparatively unrealistically biased than their Canadian counterparts. Perhaps the particular COVID-19 pandemic context gives rise to biases which are less heterogeneous across country and culture. 

Whilst patterns of comparative unrealistic optimism generally followed a similar linear trend across countries over time, levels thereof differed somewhat among European countries. For instance, Italian participants were much more likely to display comparative unrealistic optimism than their Swiss counterparts. French and British participants were almost equally likely to be more comparatively unrealistically optimistically biased than the Swiss participants, however not to the same extent as the Italians. This is surprising, given that of the four countries at the time of the surveys, Italy had been the most affected by the novel coronavirus, followed by France and the UK. It would appear from our results that the more dire the health burden from SARS-CoV-2 was for a particular country, the more likely people were to be comparatively unrealistically optimistic.

### 4.2. Potential Explanations for Paradoxical Findings

Explanations for our findings that participants became increasingly accurate in estimating their personal risk of COVID-19 infection, yet nonetheless increasingly believed others to be more at risk, warrant investigation. Despite following expert recommendations to use succinct questions soliciting independent risk estimates (e.g., 22,51]), it is possible that our results may have been influenced by the experimental design. Levels of optimism bias have been shown to be affected by the manner and order in which questions are asked [22], as well as the part of the question to which participants attended [26]. Our survey question order may have influenced participant responses; however, this is nonetheless outweighed by the external validity afforded by the current COVID-19 pandemic. Even critics of optimism bias (e.g. [115,116]), who argue that it may arise from flawed empirical methodologies, warn that any mathematical model of risk appraisal is inherently confounded by epidemic contextual factors, highlighting the urgent need for further research to be undertaken in an epidemic setting.

Experimental considerations aside, it is most likely that psychological processes, cognitive, motivational, affective, or a combination of all three, may best explain our results. One such cognitive process, or bias, may arise from challenges in interpreting numerical data, such as in processing percentages (e.g., [21,23,53,55,56]). Since the early stage of the pandemic, participants had constantly been exposed to media coverage of daily percentage SARS-CoV-2 infection updates, and may have responded by quoting the figures they had heard and read in the press, which might account for their increasingly accurate personal risk estimates. It does not, however, explain participants’ overestimation of risk for others. 

The observed comparative unrealistic optimism may have been a manifestation of a normalcy bias, a belief that had participants and their immediate social entourage managed to avoid COVID-19 up until the pandemic peak, they would likely continue to do so (e.g., [117]). Although case and hospitalisation numbers had increased exponentially, a large majority of participants knew no one in their social networks (family, friends or colleagues), who had contracted the disease [118]. As is the case for chronic disease, experience, either direct or indirect, has been found to mitigate levels of unrealistic optimism [23,34,45]. Without such direct experience, it would follow that people may have been increasingly more unrealistically optimistic for themselves, believing high levels of infection concerned others, those outside their immediate social group. Such findings are consistent with those observed in research into a large epidemic of mosquito-borne disease [61].

It is also possible that risk judgements involve two distinct cognitive processes [119]: one for ‘others’, influenced by an availability heuristic, a mental shortcut whereby importance is attributed to recently-acquired information [120], such as extensive COVID-19 media coverage, and another pertaining to the personal and immediate social sphere (i.e., family and friends)—a social circle heuristic, whereby risk estimates are based on knowledge of cases among immediate family and friends [121,122]. Optimism bias is reduced when people think objectively (epidemiologically-speaking) about their personal risk of experiencing a negative event [43,123]. Participants may have evaluated their personal risk objectively, according to reported statistics or infection rates, thereby reducing absolute optimism. Whereas participants knew their own personal health status (e.g., medical history, pre-existing conditions, protective measures), such information regarding others was unavailable, perhaps leading to subjective perceptions of greater risk and therefore comparative unrealistic optimism. 

Further support for two distinct, self/other cognitive risk appraisal mechanisms may lie in the dynamic cognitive process of belief updating. Sharot [35,124] contends that unrealistic optimism results from positive belief updating, or the preferential integration of positive rather than negative information, so as to reinforce a positive self-image. Although compelling, this only explains our results regarding comparative unrealistic optimism and not the observed trend in absolute unrealistic optimism. In contrast to Sharot [35,124], Shah et al. [116], using Bayesian analysis, found evidence for baseline belief updating in both positive and negative directions. This seems to be supported by our study in which participants appear to update baseline information they hold regarding the risk to themselves and familiar others, to become less absolutely optimistic over time, whilst simultaneously updating their baseline appraisal of risk for others according to new, updated information obtained via media portrayals of ‘others’, to become increasingly comparatively unrealistically optimistic over time. 

Explanations for the increasing levels of comparative unrealistic optimism may extend beyond the domain of cognition to motivation and affect [125]. Affect aroused by the COVID-19 threat may have motivated participants to downplay their relative risk. Slovic, Finucane, Peters and MacGregor [126], following from Zajonc [127], proposed that an ‘affect heuristic’ may shape risk judgements. Lau et al. [71] contend that circumstances specific to an unfolding epidemic of a highly contagious disease of serious or fatal consequences contribute to optimism bias. Indeed, Park, Ju, Ohs and Hinsley [128] found an association between affect and unrealistic optimism in COVID-19 risk judgements. This could explain our paradoxical results. As participants adjusted to the very real threat posed by this new coronavirus (reflected in decreasing absolute optimism scores), emotional and affective reactions, or defence mechanisms, may have served to downplay or deny the risk for oneself, resulting in comparative unrealistic optimism. Such denial or defensive distortion has been observed in situations where it is not possible to reduce the threat [129,130,131], as was the case during the first wave of the COVID-19 pandemic. As Slovic et al. [126] so eloquently state: “in some decision-making circumstances, reliance on affect and emotion is a quicker, easier and more efficient way to navigate in a complex, uncertain and sometimes dangerous world” [126] (p. 1334). The affect aroused by the COVID-19 threat may inspire a defensive response of reassurance, whereby people rationalise that if they are at risk, then others are far more so. 

Lack of control [34,43,132], fear [23] and uncertainty [67] have been observed to contribute to unrealistic optimism, and may even be healthy in an uncertain situation [35,40,124], as a direct, self-regulatory response to adversity, fear and a need for control [133,134,135]. Fear and anxiety towards COVID-19 have been strongly associated with comparative unrealistic optimism [114,136]. Constant media transmission of disturbing images of critically-ill patients fighting for their lives at the mercy of a new, poorly understood, life-threatening respiratory disease may have elicited an emotional, defensive, distancing reaction that influenced comparative risk judgements. The novel nature of this virus, its rapid, uncontrolled proliferation and often severe consequences, no doubt also contributed to this affect-laden response and consequent optimism bias [2,3,21,43,71]. The affect arising from the COVID-19 pandemic may mitigate any rational risk appraisal, transforming a rational cognitive personal judgement into an affect-laden reactional comparative one.

### 4.3. Comparative Optimism and Health-Protective Behaviours

Comparative unrealistic optimism towards chronic health issues has long been associated with a reduction in engagement in protective behaviours (e.g., [22,41,97]). Understanding the complex interplay between risk appraisal and behaviour in a pandemic context is therefore critical to combatting the spread of novel health threats, such as that posed by COVID-19, reliant upon individual adoption of preventive measures.

Unlike Xu and Peng’s findings that the association between optimism bias and behaviour was unstable over time [68], our research, as did that of Fragkaki et al. [136] and Wise et al. [3], found unrealistic optimism to be negatively associated with the majority of protective behaviours examined. People who were comparatively unrealistically optimistically biased were less likely than realists or pessimists to engage in protective behaviours such as wearing a face mask, avoiding touching their mouth and nose or using sanitising hand gel, perhaps because they perceived themselves to be less at risk. 

Our results appear to be largely influenced by contextual factors. Health authorities in the various countries at that stage advised against wearing masks [87]; however, this does not explain why realists and pessimists were significantly more likely than unrealistic optimists to wear masks. Perhaps unrealistic optimists nonetheless perceived themselves to be less at risk. The lack of engagement by unrealistic optimists in using sanitising hand gel or avoiding touching one’s nose and mouth is surprising, as these measures were promoted in nationwide public health campaigns across Europe [14,89], and is perhaps a manifestation of comparative risk underestimation. If mask-wearing were considered as an altruistic act designed to reduce risk for others, unrealistic optimists’ mask reluctance evokes a worrying, rather individualistic stance, given the rapid pandemic spread. 

During the third survey, all four countries were in lockdown (full restrictions in Switzerland). Government statistics suggest that this impacted approximately 70% of the population, with approximately 30% continuing to travel to work [137,138,139,140,141,142,143]. With over two-thirds of people confined, this context may have reduced the need for protective measures, thereby contributing to participants’ high, and perhaps justifiable, levels of optimism bias. Indeed, adopting measures, such as using hand sanitiser and wearing a mask, whilst necessary when engaging in “normal” professional and social activities, is greatly reduced in confinement.

People’s being in lockdown may have influenced comparative risk appraisals in an unforeseen way. Being physically and socially distanced, participants had no way of knowing whether others were respecting confinement or barrier actions. Thanks to media portrayals of people flaunting lockdown rules, others appeared less cautious and possibly judged more at risk of infection.

Interestingly, only three protective measures were adopted to a similar extent by unrealistic optimists, pessimists and realists: frequent hand washing, single-usage of a tissue when coughing or sneezing and avoiding public transport. Unrealistic optimists’ engagement in the first two measures, clearly visible to others, may have been motivated more by social desirability, a wish to be viewed favourably by others, rather than in response to perceived risk. Perhaps in this particular pandemic setting, frequent hand washing and once-only-tissue-usage became a social norm. Avoiding public transport was possible for those in lockdown. Even for the remaining 30%, for whom it was necessary to travel to work, alternatives to public transport (e.g., bike, car, electric scooter) were available. 

Previous investigations of comparative unrealistic optimism posited that as a result of engagement in protective behaviours, people become optimistic that they will not encounter the particular health threat [22]. At first glance, this is not reflected in the current study. Researchers warn that it is unclear whether optimism bias is a cause or a consequence of engaging in protective behaviour [32,43,51]. It is possible that unrealistic optimism was a consequence of the mandatory protective measure of physical distancing during lockdown. Whether cause or consequence, the pandemic context clearly contributes to levels of unrealistic optimism, and merits greater attention in future research and public health campaigns. 

Conducted in an evolving pandemic setting, this study provides a unique insight into the complex relationship between epidemiological context, risk appraisal and protective behaviour. The evolutive coronavirus pandemic setting and the affect it generates no doubt have an impact upon the cognitive processes involved in risk appraisal and subsequent decisions to engage in protective measures. At present, the precise way in which affect interacts with cognition (and cognitive biases) remains unknown. This research in situ, as the COVID-19 pandemic raged across Europe, sheds light on the role played by risk appraisal in the adoption of and adherence to protective measures as a pandemic unfolds and takes into consideration the dynamic nature of a constantly changing epidemic context. It is hoped that our findings will not only contribute to informing future public health communication and campaigns, but also pave the way for further research in this crucial public health area, critical in combatting twenty-first-century health threats. 

### 4.4. Limitations

Despite its ecological strength in being conducted during the unfolding 2020 COVID-19 pandemic, the current study nonetheless has several limitations. It is cross-sectional and although it extends across four European countries, there is no control group in this pandemic. Were one to discover such a group, withholding information pertaining to the severity and contagiousness of COVID-19, as well as relevant protective barrier measures, would be questionable at best, if not downright unethical. 

It is possible that a self–selection bias may be operating in that people who participate in the “Influenzanet” survey may already be interested in, informed and concerned about influenza-like illnesses and therefore they may not be representative of the general population. Another selection bias may have occurred for the 65+ age group which, despite being at risk of developing more severe forms of the disease, may be under-represented due to difficulties with computer-literacy. 

A further limitation of this study lies in the measurement of comparative unrealistic optimism. Other measurements of unrealistic optimism could have been used, such as that in which it is calculated as a difference between “me” and “peer”. However, our means of measuring unrealistic optimism has been adopted by many before us (e.g., [24]) and was therefore deemed appropriate by all four participating countries. These limitations are nonetheless compensated by the extremely high ecological validity and relevance afforded by the government surveillance body data, gathered under pandemic conditions. To date, prior research in this area has considered at best two epidemic stages—pre- and peak (e.g., [3,21,68])—and has predominantly involved a hypothetical epidemic outbreak. The current study, conducted during an epidemic outbreak of pandemic proportions, makes a valuable contribution to research. 

## 5. Conclusions

This research into the dynamic nature of unrealistic optimism and health-protective behaviour across four European countries during an unfolding, rapidly evolving pandemic makes a unique contribution to our understanding of risk perception in an uncertain environment. The paradoxical decrease in absolute unrealistic optimism, as comparative unrealistic optimism increased, shows that the magnitude of unrealistic optimism depends to a large extent on when individuals are asked about infection risk. From our findings, it is clear that levels of absolute and comparative unrealistic optimism, as well as protective behaviour, were associated with and influenced by the particular stage of the epidemic. It was within this very affect-laden context, as the pandemic raged to its peak in Europe, that people made risk judgements that determined their adoption or otherwise of protective measures which, in turn, had an impact on the spread and health burden of COVID-19. Indeed, the nationwide, government-imposed, collective preventive measures no doubt had a further impact upon risk appraisal and behaviour. Future research into risk appraisal and behavioural response in an epidemic may reveal whether similar patterns of unrealistic optimism and behaviour are observed. In particular, it may be relevant to explore how two distinct cognitive mechanisms, an availability heuristic and a social circle heuristic, contribute to personal and general risk estimates in response to varying risk environments. Gaining insight into the complex interplay between epidemic context, unrealistic optimism and protective behaviour would help better inform public health prevention campaigns, critical to reducing disease spread and the health burden of novel, unpredictable, emerging infectious diseases, such as COVID-19. 

## Figures and Tables

**Table 1:** Measures: survey questions and answers.

Survey Question	Possible Answers							
**Risk Perception**								
What is the risk for yourself of catching this coronavirus in the coming weeks?	0%	0–1%	1–3%	3–5%	5–10%	10–20%	20–50%	>50%
In your opinion, out of 100 [name of country] people, how many of them are at risk of catching this coronavirus in the coming weeks?	0%	0–1%	1–3%	3–5%	5–10%	10–20%	20–50%	>50%
I think I already had this disease. [Survey 3 only]	Yes	No						
**Protective Behaviours**								
Which of the following do you do as a result of this coronavirus?								
Wash your hands often	Yes	No						
Wear a face mask	Yes	No						
Avoid touching your mouth or nose	Yes	No						
Use a tissue only once when coughing or sneezing	Yes	No						
Avoid public transportation	Yes	No						
Use sanitizing hand gel	Yes	No						
Avoid contact with people who look sick	Yes	No						
Avoid social events	Yes	No						

**Table 2:** Distribution of optimism, realism and pessimism related to SARS-CoV-2 infection across countries and epidemic stages.

Risk Perception		Survey 1	Survey 2	Survey 3	*C*^2^ (*df*), *p*-Value
Italy	Optimism	47.4% (120)	59.2% (155)	74.6% (296)	104 (4), <10^−5^
	Realism	47.0% (119)	26.3% (69)	11.8% (47)	
	Pessimism	5.5% (14)	14.5% (38)	13.6% (54)	
France	Optimism	55.4% (1880)	38.7% (1012)	67.2% (2536)	927 (4), <10^−5^
	Realism	39.2% (1329)	42.5% (1110)	16.2% (611)	
	Pessimism	5.4% (184)	18.8% (492)	16.6% (627)	
Switzerland	Optimism	40.8% (158)	39.5% (92)	61.8% (252)	96 (4), <10^−5^
	Realism	9.3% (36)	42.1% (98)	18.6% (76)	
	Pessimism	49.9% (193)	18.5% (43)	19.6% (80)	
United Kingdom	Optimism	39.4% (54)	44.8% (107)	72.2% (200)	85 (4), <10^−5^
	Realism	56.2% (77)	40.6% (97)	15.5% (43)	
	Pessimism	4.4% (6)	14.6% (35)	12.3% (34)	
All	Optimism	53.0% (2213)	40.8% (1366)	67.2% (2536)	1135 (4), <10^−5^
	Realism	41.2% (1718)	41.0% (1374)	16.6% (627)	
	Pessimism	5.8% (241)	18.2% (608)	16.2% (611)	

**Table 3:** Distribution of optimism, realism and pessimism related to SARS-CoV-2 infection in a variety of subpopulations and settings: numbers and percentages.

Variable		Optimism %(*N*)	Realism %(*N*)	Pessimism %(*N*)	*C*^2^ (*df*), *p*-Value
Sex	Male	50.9% (3013)	14.8% (874)	34.3% (2031)	95 (2), <10^−5^
	Female	59.6% (3851)	11.9% (770)	28.5% (1838)	
Age group	18–35	50.5% (1622)	14.5% (465)	35.1% (1127)	63 (4), <10^−5^
	35–65	56.1% (3481)	12.6% (779)	31.4% (1946)	
	65 and older	59.6% (1761)	13.5% (400)	26.9% (796)	
Occupation	Employed	51.9% (3433)	13.7% (907)	34.4% (2280)	96 (6), <10^−5^
	Unemployed	64.9% (674)	10.9% (113)	24.2% (251)	
	Retired	58.4% (2349)	13.1% (526)	28.6% (1149)	
	Student	58.7% (273)	14.6% (68)	26.7% (124)	
	Other	59% (135)	12.7% (29)	28.4% (65)	
Education	Some high school	58.2% (1647)	15.1% (428)	26.7% (757)	102 (4), <10^−5^
	High school	58.7% (2690)	12.2% (560)	29.1% (1331)	
	Some college and higher	50.9% (2527)	13.2% (655)	35.9% (1781)	
Composition of household	Living with one or several children	54.7% (1616)	14.7% (433)	30.6% (904)	6 (4), 0.18
	Living with other adults but no child	55.5% (3935)	12.9% (913)	31.6% (2239)	
	Living alone	55.9% (1288)	12.9% (298)	31.2% (718)	
	Missing data	74.3% (26)	2.9% (1)	22.9% (8)	
Health status	No chronic health condition	55.5% (5228)	12.4% (1173)	32.1% (3025)	30 (2), <10^−5^
	Chronic health condition	55.4% (1635)	16% (471)	28.6% (844)	
Country	Switzerland	48.8% (502)	15.5% (160)	35.7% (367)	40 (6), <10^−5^
	France	55.5% (5429)	13.3% (1303)	31.2% (3051)	
	Italy	62.7% (572)	11.6% (106)	25.7% (235)	
	United Kingdom	55.3% (361)	11.5% (75)	33.2% (217)	
Survey	Pre-epidemic stage	53% (2213)	5.8% (241)	41.2% (1718)	1131 (4), <10^−5^
	Early epidemic stage	40.8% (1366)	18.2% (608)	41% (1374)	
	Epidemic peak stage	67.6% (3285)	16.4% (795)	16% (777)	

**Table 4:** Personal and contextual determinants of unrealistic optimism related to SARS-CoV-2 infection: unadjusted and adjusted odds ratio (UOR, AOR), 95% confidence interval, and *p*-value.

Variable		UOR (95% CI)	*p*-Value	AOR (95% CI)	*p*-Value
Sex	Male	Ref.		Ref.	
	Female	1.39 [1.27;1.51]	<0.001	1.4 [1.29;1.52]	<0.001
Age group	18–35	Ref.		Ref.	
	35–65	1.10 [0.94;1.28]	0.050	1.09 [0.93;1.27]	0.048
	>65	1.28 [1.04;1.58]	0.050	1.27 [1.04;1.57]	0.048
Occupation	Employed	Ref.		Ref.	
	Unemployed	1.32 [1.11;1.57]	0.003	1.3 [1.1;1.55]	0.003
	Retired	1.18 [1.01;1.36]	0.003	1.17 [1.02;1.35]	0.003
	Student	1.28 [0.91;1.82]	0.003	1.29 [0.92;1.83]	0.003
Education	High school	Ref.		Ref.	
	Some high school	1.11 [0.95;1.29]	0.001	1.1 [0.95;1.28]	<0.001
	Some college and higher	0.89 [0.8;0.99]	0.001	0.88 [0.79;0.98	<0.001
Stage	Pre-epidemic	Ref.		Ref.	
	Early epidemic	0.76 [0.69;0.83]	<0.001	0.76 [0.69;0.84]	<0.001
	Epidemic peak	2.38 [2.17;2.61]	<0.001	2.38 [2.17;2.61]	<0.001
Country	Switzerland	Ref.		Ref.	
	France	1.29 [1.11;1.49]	<0.001	1.29 [1.11;1.49]	<0.001
	Italy	1.81 [1.47;2.22]	<0.001	1.82 [1.48;2.23]	<0.001
	United Kingdom	1.21 [0.97;1.51]	<0.001	1.22 [0.98;1.53]	<0.001
Composition of household	Living with other adult(s), without a child	Ref.			
	Living alone	0.97 [0.87;1.09]	0.607		
	Living with one or several children	0.95 [0.85;1.05]	0.607		
Health status	No chronic health condition	Ref.			
	Chronic health condition	0.92 [0.84;1.01]	0.097		

**Table 5:** Association between unrealistic optimism and adoption of health-protective behaviours recommended to control the risk of infection by SARS-CoV-2: odds ratio and 95% CI (adjusted for sex, age, education, and country), *p*-value.

Health Behaviour	AOR and 95% CI	*p*-Value
Wash hands often	0.89 [0.77;1.03]	0.13
Wear face mask	0.65 [0.54;0.78]	<0.0001
Avoid touching one’s mouth and nose	0.86 [0.75;0.99]	0.041
Use a unique tissue when coughing or sneezing	0.9 [0.79;1.03]	0.12
Avoid public transportation	1.1 [0.96;1.26]	0.16
Use sanitising hand gel	0.75 [0.67;0.86]	<0.0001
Avoid contact with people who look sick	1.15 [1.01;1.31]	0.036

## Data Availability

The data presented in this study are available on request from Dr Marion Debin (marion.debin@iplesp.upmc.fr). The data are not publicly available due to compliance with the European regulation about personal data protection (RGPD).
